# Challenges facing the elderly care industry in Hong Kong: the shortage of frontline workers

**DOI:** 10.1186/2193-1801-3-S1-P1

**Published:** 2014-12-04

**Authors:** Stella, Sin-tung Kwok, Kris, Wai-ning Wong, Shun-lai Yang

**Affiliations:** Research Support Unit, Vocational Training Council, Kragujevac, Hong Kong; Li Ka Shing Institute of Professional and Continuing Education, The Open University of Hong Kong, Kragujevac, Hong Kong; Supportive Work—team for Active-ageing Network, Vocational Training Council, Kragujevac, Hong Kong

## Background

Ageing is a major population problem worldwide, including Hong Kong. With better medical technology, people typically live longer. In 2012, the life expectancy for men and women in Hong Kong was 81 and 86 years, respectively, an increase of 8 years compared with 30 years ago [1]. However, despite a longer lifespan, many elderly people are living with chronic illnesses, creating a huge demand for elderly services. The Hong Kong Government introduced the concept of ‘Ageing in Place’ in 2007. This latest policy paper on ageing explicitly encourages the elderly to stay at home and in the community [2, 3]. Although such a policy seems reasonable on paper, its implementation hinges on many associated supportive policies and services. Even though the number of elderly service providers has increased over the past few years, the demand still far exceeds the supply. In fact, elderly service providers are facing increasing difficulty to keep pace with the mass demand for their services. With such high demand and limited funding being allocated, elderly service workers are often forced to overwork but are underpaid and experience high work stress [4]. Elders, in turn, are being put at risk for substandard services caused by insufficient funding, mismatch of benefits allocation, the lack of manpower, highly stressed staff and much more. The present study was conducted to examine the recruitment challenges Hong Kong’s elderly care service providers are facing and their perceptions of the HKSAR government’s latest elderly policy, ‘Ageing in Place’, and the role of vocational education in training manpower for the industry.

## Methods

A survey entitled ‘Challenges of Elderly Service Industry in Hong Kong’ was developed according to the results of a previously conducted qualitative study[5]. The questionnaire consisted of 26 questions aiming to gather demographic characteristics of participants, their routine operational procedures, their opinions on the government’s policy of ‘Ageing in Place’ and their perception of vocational education and training. All registered elderly service providers listed with the Social Welfare Department of Hong Kong were invited to participate in this survey. Of all 551 elderly organizations and service units consulted, 197 (35.8%) completed the survey for analysis.

## Results

Most of the respondents believed that insufficient remuneration (74.1%) and poor career prospects (71.6%) are the main reasons for causing difficulty when hiring frontline staff in promoting or facilitating active aging. The majority of respondents believed that vocational education and on-the-job training are effective models and could help to maintain a stable supply of frontline care workers. Young people should also be provided with reliable and impartial sources of career guidance so that there is less chance for them to choose the wrong career. Apart from that, supportive social services, coupled with easy transportation, affordable housing for independent living, policies, public education and initiatives on promoting inter-generational harmony are also required to sustainably implement ‘active ageing’. Results of the present study echo another recent qualitative study conducted by Wong et al. [5], which identified recruitment difficulty, government policy fragmentation and funding rigidity as the three major challenges that raised the most attention of elderly service providers.

Recruitment difficulty, especially of frontline care- and non-care-oriented workers, was perceived to be the major challenge, and such difficulty was reported to be closely related to and be influenced by government policy and the availability of funding. All respondents agreed that vocational education and training (VET) might be one of the solutions for effective implementation of ‘Ageing in Place’.

## Conclusions

VET providers, elderly service providers and enterprises could join hands to provide timely and relevant education and training programs targeted to attract and retain talent for the elderly care industry.
